# Supplementary material for: Intranasal Replicating Adenovirus type 4-SARS-CoV-2 Recombinants Induce Superior Immune Response Durability and Efficacy in Preclinical Testing Compared to Standard Intramuscular Vaccines
Source: bioRxiv. 2026 Feb 5:2026.02.04.703921. Preprint. [Version 1] doi: 10.64898/2026.02.04.703921 (PMC13119332; doi:10.64898/2026.02.04.703921)
Supplement: 1 [file NIHPP2026.02.04.703921v1-supplement-1.pdf]

bioRxiv preprint doi: https://doi.org/10.64898/2026.02.04.703921; this version posted February 5, 2026. The copyright holder for this preprint (which was not certified by peer review) is the author/funder. This article is a US Government work. It is not subject to copyright under 17 USC 105 and is also made available for use under a CC0 license.

143

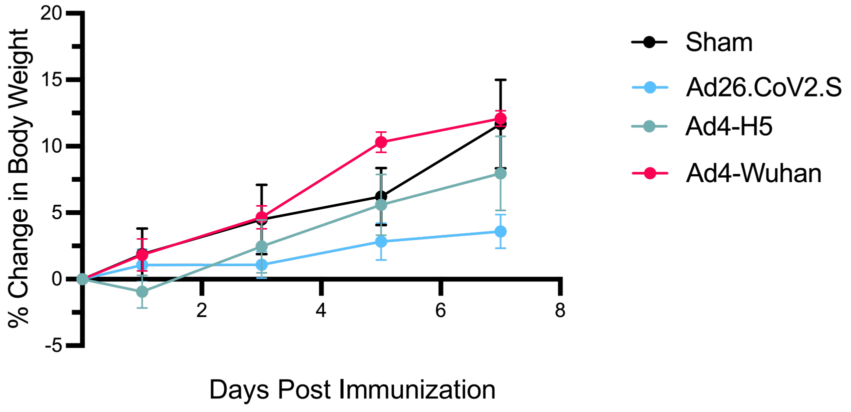

Supplementary Figure 1. Immunization with Ad4-constructs alone does not significantly impact hamster weight. Longitudinal weight change of hamsters administered IM sham, IM Ad26.CoV2.S, IN Ad4-H5, or Ad4-Wuhan.

bioRxiv preprint doi: https://doi.org/10.64898/2026.02.04.703921; this version posted February 5, 2026. The copyright holder for this preprint (which was not certified by peer review) is the author/funder. This article is a US Government work. It is not subject to copyright under 17 USC 105 and is also made available for use under a CC0 license.

223

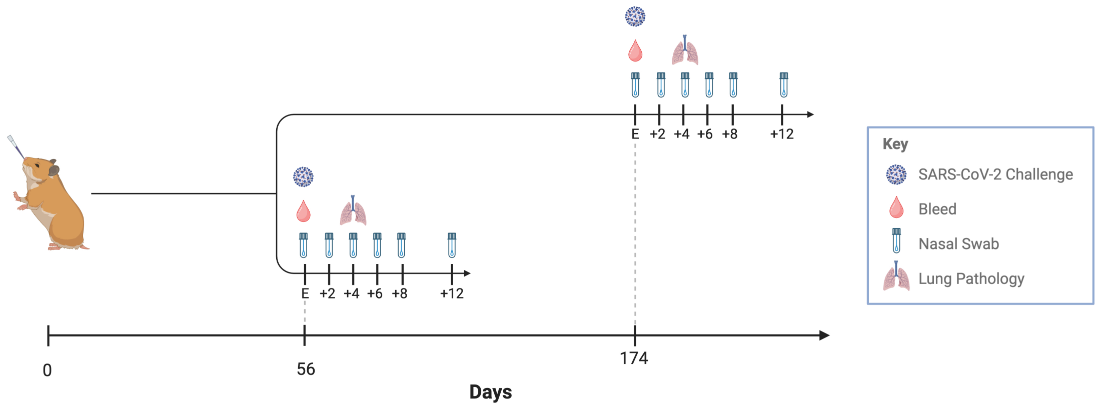

Supplementary Figure 2. Challenge study outline. Hamsters received IN or IM vaccines on day 0 and then challenged on day 56 or day 174 with either SARS-CoV-2 Delta or Omicron. Samples were then taken at the indicated timepoints.

bioRxiv preprint doi: https://doi.org/10.64898/2026.02.04.703921; this version posted February 5, 2026. The copyright holder for this preprint (which was not certified by peer review) is the author/funder. This article is a US Government work. It is not subject to copyright under 17 USC 105 and is also made available for use under a CC0 license.

233

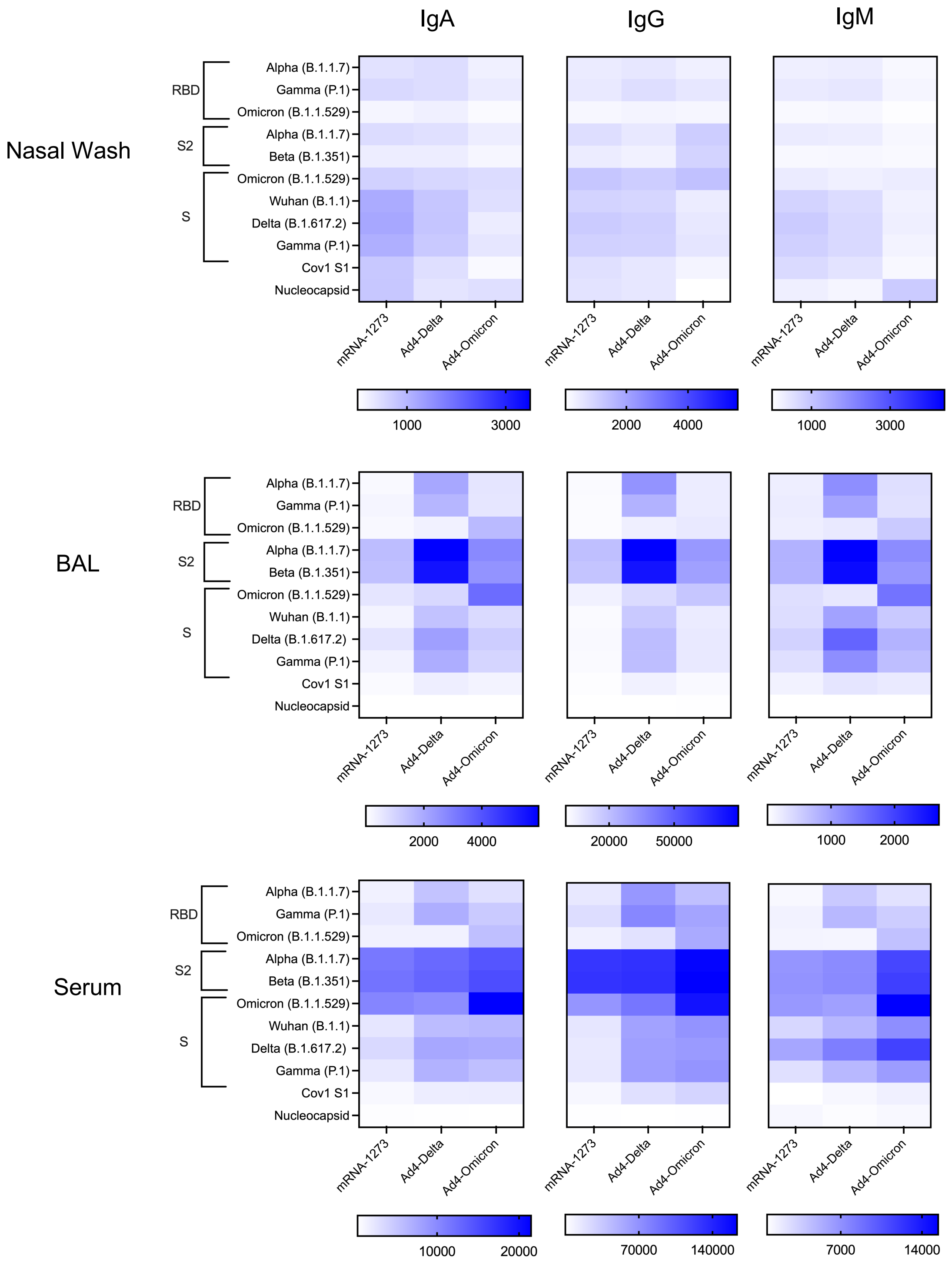

Supplementary Figure 3. IN Ad4-Spike binding antibody responses from the D180 nasal mucosa. Binding Ig characterization in hamster nasal wash, BAL, and serum against the indicated SARS-CoV antigens, including Spike (S) and Spike subunits, the receptor binding domain (RBD), and Nucleocapsid proteins.

bioRxiv preprint doi: https://doi.org/10.64898/2026.02.04.703921; this version posted February 5, 2026. The copyright holder for this preprint (which was not certified by peer review) is the author/funder. This article is a US Government work. It is not subject to copyright under 17 USC 105 and is also made available for use under a CC0 license.

Serum: Ad4-Wuhan compared to 3 control groups (Sham, Ad4-H5, and Ad26.CoV.2.S)

	Ad4-Wuhan vs.								
	Sham	Ad4-H5	Ad26.CoV.2.S	Sham	Ad4-H5	Ad26.CoV.2.S	Sham	Ad4-H5	Ad26.CoV.2.S
Binding Antigens	Serum IgA			Serum IgG			Serum IgM		
Alpha RBD (B.1.1.7)	0.002	0.002	0.026	0.002	0.002	0.485	0.002	0.002	0.937
Gamma RBD (P.1)	0.002	0.002	0.065	0.002	0.002	0.937	0.004	0.002	0.180
Omicron RBD (B.1.1.529)	0.002	0.002	0.229	0.002	0.005	0.002	0.310	0.180	0.521
Alpha S2 (B.1.1.7)	0.002	0.002	0.180	0.005	0.005	0.013	0.002	0.002	0.818
Beta S2 (B.1.351)	0.002	0.002	0.041	0.002	0.002	0.015	0.002	0.002	0.818
Omicron S (B.1.1.529)	0.004	0.005	0.012	0.002	0.002	1.000	0.002	0.002	0.093
Alpha S B.1.1.7	0.005	0.005	0.005	0.002	0.002	0.002	0.002	0.002	0.004
Delta S (B.1.617.2)	0.002	0.002	0.009	0.005	0.002	0.149	0.002	0.002	0.937
Gamma S (P.1)	0.004	0.002	0.126	0.002	0.002	0.132	0.002	0.002	0.748
Cov1 S1	0.009	0.002	0.310	0.002	0.002	0.180	0.009	0.004	0.485
Nucleocapsid	0.934	0.677	1.000	0.375	0.090	0.374	0.015	0.261	0.630

BAL: Ad4-Wuhan compared to 3 control groups (Sham, Ad4-H5, and Ad26.CoV.2.S)

	Ad4-Wuhan vs.								
	Sham	Ad4-H5	Ad26.CoV.2.S	Sham	Ad4-H5	Ad26.CoV.2.S	Sham	Ad4-H5	Ad26.CoV.2.S
Binding Antigens	BAL IgA			BAL IgG			BAL IgM		
Alpha RBD (B.1.1.7)	0.005	0.005	0.589	0.004	0.004	0.180	0.005	0.045	0.240
Gamma RBD (P.1)	0.005	0.005	0.394	0.004	0.004	0.132	0.004	0.041	0.310
Omicron RBD (B.1.1.529)	0.003	0.012	0.589	0.004	0.005	0.818	0.148	0.630	0.485
Alpha S2 (B.1.1.7)	0.002	0.005	0.041	0.004	0.004	0.009	0.005	0.065	0.030
Beta S2 (B.1.351)	0.004	0.005	0.015	0.004	0.004	0.002	0.004	0.065	0.015
Omicron S (B.1.1.529)	0.003	0.004	0.016	0.003	0.004	0.004	0.002	0.009	0.065
Alpha S (B.1.1.7)	0.004	0.004	0.002	0.004	0.005	0.002	0.002	0.004	0.002
Delta S (B.1.617.2)	0.005	0.005	0.132	0.005	0.005	0.004	0.002	0.015	0.093
Gamma S (P.1)	0.004	0.004	0.378	0.004	0.005	0.394	0.002	0.013	0.394
Cov1 S1	0.037	0.029	0.589	0.002	0.005	0.937	0.092	0.936	0.575
Nucleocapsid	0.074	0.176	0.176	0.809	0.935	0.172	1.000	0.568	1.000

NW: Ad4-Wuhan compared to 3 control groups (Sham, Ad4-H5, and Ad26.CoV.2.S)

bioRxiv preprint doi: https://doi.org/10.64898/2026.02.04.703921; this version posted February 5, 2026. The copyright holder for this preprint (which was not certified by peer review) is the author/funder. This article is a US Government work. It is not subject to copyright under 17 USC 105 and is also made available for use under a CC0 license.

	Ad4-Wuhan vs.								
	Sham	Ad4-H5	Ad26.CoV.2.S	Sham	Ad4-H5	Ad26.CoV.2.S	Sham	Ad4-H5	Ad26.CoV.2.S
Binding Antigens	NW IgA			NW IgG			NW IgM		
Alpha RBD (B.1.1.7)	0.015	0.005	0.005	0.018	0.004	0.012	0.004	0.004	0.002
Gamma RBD (P.1)	0.008	0.005	0.002	0.004	0.004	0.026	0.008	0.005	0.005
Omicron RBD (B.1.1.529)	0.087	0.006	0.006	0.121	0.103	1.000	0.394	0.173	0.394
Alpha S2 (B.1.1.7)	0.005	0.005	0.005	0.005	0.004	0.005	0.004	0.005	0.005
Beta S2 (B.1.351)	0.005	0.003	0.005	0.005	0.005	0.005	0.005	0.004	0.005
Omicron S (B.1.1.529)	0.004	0.005	0.009	0.002	0.007	0.041	0.180	0.026	0.041
Alpha S B.1.1.7	0.012	0.005	0.004	0.004	0.005	0.005	0.005	0.004	0.002
Delta S (B.1.617.2)	0.013	0.005	0.002	0.004	0.005	0.019	0.009	0.004	0.008
Gamma S (P.1)	0.029	0.012	0.004	0.026	0.050	0.056	0.015	0.026	0.041
Cov1 S1	0.261	0.017	0.180	0.463	0.810	0.574	0.267	0.200	0.518
Nucleocapsid	0.797	1.000	1.000	0.936	0.463	0.560	0.467	0.295	0.196
